# Cancer-Derived Extracellular Vesicles: Their Role in Sarcoma

**DOI:** 10.3390/life12040481

**Published:** 2022-03-26

**Authors:** Anita Adib, Ruhi Sahu, Shivangi Mohta, Raphael Etomar Pollock, Lucia Casadei

**Affiliations:** 1 The James Cancer Hospital Solove Research Institute, The Ohio State University, Columbus, OH 43210, USA; mohta.9@buckeyemail.osu.edu (S.M.); lucia.casadei@osumc.edu (L.C.); 2 Division of Surgical Oncology, Department of Surgery, The Ohio State University, Columbus, OH 43210, USA; raphael.pollock@osumc.edu

**Keywords:** extracellular vesicles, sarcoma, cargo, cancer, Ewing’s sarcoma, rhabdomyosarcoma, Kaposi’s sarcoma, osteosarcoma, liposarcoma, tumorigenesis

## Abstract

Soft tissue sarcomas (STS) are rare malignancies with limited responses to anticancer therapy. Extracellular vesicles (EVs) are a heterogeneous group of bi-lipid layer sacs secreted by cells into extracellular space. Investigations of tumor-derived EVs have revealed their functional capabilities, including cell-to-cell communication and their impact on tumorigenesis, progression, and metastasis; however information on the roles of EVs in sarcoma is currently limited. In this review we investigate the role of various EV cargos in sarcoma and the mechanisms by which those cargos can affect the recipient cell phenotype and the aggressivity of the tumor itself. The study of EVs in sarcoma may help establish novel therapeutic approaches that target specific sarcoma subtypes or biologies, thereby improving sarcoma therapeutics in the future.

## 1. Introduction

### 1.1. What Are Extracellular Vesicles?

Extracellular vesicles (EVs) are bi-lipid layer sacs secreted by the cell into the extracellular space [1]. EVs have many roles, including participating in cell-cell communication and cell maintenance [1]. They have the ability to actively package cellular contents, including proteins, nucleic acids, and lipids. These functional cargos can be then delivered to other cells with discrete effects in recipients.

Recent studies have shown that cancer cells can use EVs to enhance tumor progression and metastasis; therefore, EVs have become the focus of study in cancer. However, only a small number of reports have addressed the question of EV involvement in sarcoma pathobiology, the focus of this review.

### 1.2. Mechanisms of Formation, Release, and Uptake

Despite the lack of full comprehension of EV formation, release, and uptake molecular mechanisms, studies have shown that various signaling molecules tightly regulate these controlled processes [2]. Plasma membranes have the ability to bud both inward and outward to form membrane-encapsulated vesicles due to their versatile structure [3].

Small extracellular vesicles are found to be generated by the inward budding of multivesicular bodies (MVBs) which result in the formation of intraluminal vesicles (ILVs) [4]. After endosome maturation, the MVBs fuse with the plasma membrane and liberate the enclosed ILVs, which are now referred to as EVs when in the extracellular space [4]. The biogenesis of MVBs involves multiple potential mechanisms; i.e., lipid raft microdomains [4,5]. While sEV biogenesis may depend on endosome membrane characteristics and sorting of cargo molecules, three pathways have been noted to mediate biogenesis: the endosomal sorting complex required for transport (ESCRT) pathway, the ceramide dependent pathway, and the tetraspanin dependent pathway [2,4].

ESCRT-dependent mechanisms have been widely studied as a mediator of MVB biogenesis by sorting ubiquitinated proteins into ILVs [4,6]. ESCRT-0, -I, -II, and -III along with accessory proteins, such as ALIX and VTA1, form multiprotein complexes that concentrate on the cytoplasmic portion of the endosomal membrane. These complexes regulate MVB and sEV formation and release [7]. The pathway is initiated by ESCRT-0, which maintains ubiquitinated proteins in the late endosomal membrane [6]. ESCRT-I and ESCRT-II bind cargo, ESCRT-II nucleates ESCRT-III, ESCRT-III separates vesicles, and accessory proteins assist in recycling the components [6,8]. It remains unclear whether MVBs produced by such ubiquitin-dependent mechanisms fuse with lysosomes to be degraded with their contents or if they fuse with the plasma membrane to release their contents into the extracellular space as EVs [2,4].

ESCRT-independent mechanisms, such as the ceramide-dependent pathway and tetraspanin-dependent pathway, are also involved in sEV biogenesis involving lipids, tetraspanins, or heat shock proteins [8]. One study investigating oligodendroglial cells found that the inhibition of neural sphingomyelin impeded ceramide biogenesis and thus reduced EV secretion [5]. Sphingomyelin hydrolysis forms ceramide, which was proposed to prompt MVBs to invaginate and form ILVs [5]. Other studies have proposed that tetraspanin proteins may be involved in selecting cargo for EV secretion [8]. A study investigating melanosomes found that CD63 engages in sorting of PMEL into ILVs without the use of ceramide or ESCRT complexes [9]. As discoveries are made regarding EV sorting mechanisms, further research is needed to understand how different sorting mechanisms affect the subtype or makeup of EVs released by cancer cells [4].

Once MVBs mature, they either degrade their contents by fusing with lysosomes or release their contents as EVs by fusing with the plasma membrane [4]. However, the differences between the two types of MVBs remain unclear [4]. While there is little to no consensus in the areas of EV storage and stability, recent studies have shown that the uptake of EVs by recipient cells may depend on interactions between surface receptors and ligands, direct fusion, endocytosis, and cargo transferred [2]. Kinesin transports MVBs undergoing exocytosis to the plasma membrane [4]. Proteins in the EV membrane, such as RAB GTPase proteins and RAB effector molecules, control vesicle budding, vesicle docking, and intracellular vesicular tracking to regulate EV secretion [2,8]. For instance, RAB11 and RAB35 mediate recycling endosomes, while RAB7 mediates the maturation of late endosomes [10]. Accordingly, RABs may be involved in different stages of the endosomal pathway; MVBs could potentially give rise to different sEV subtypes [8]. Once the intracellular components are transported and docked to the plasma membrane, MVBs pair with soluble N-ethylmaleimide-sensitive factor (NSF)-attachment protein receptor (SNARE) complexes for lipid bilayer fusion which is controlled by many regulatory mechanisms [4]. After the MVBs successfully fuse with the plasma membrane, EVs are released into the extracellular space to influence recipient cell function, interact with the extracellular matrix, or enter circulation [4].

In addition to MVB-derived EVs, EVs can also form from the outward budding of cell plasma membranes and are typically deemed large EVs or microvesicles [11]. Studies have proposed that this process involves cytoskeleton components, SNAREs, and tethering factors [11,12]. The uptake of lEVs is lower at lower temperatures and is likely an energy-dependent process [11]. lEVs are very heterogeneous in size, suggesting the possibility of multiple potential mechanisms for lEV biogenesis [4]. One such mechanism that suggests plasma membrane origin involves the endosomal sorting complex required for transport (ESCRT) machinery to produce EVs enriched in cell surface proteins [3,4]. Acid sphingomyelinase may likewise play a role in EV biogenesis by promoting ceramide-dependent lEV production [4,13].

### 1.3. EV Subtypes: sEV and lEV

Extracellular vesicle (EV) is the generic term describing cell-originating released particles containing a lipid bilayer and lacking the ability to replicate [14]. EVs are usually categorized based on their origin, biogenesis pathway, associations or function [2]. Regardless of origin, all EVs contain biomolecules, e.g., DNA, RNA, proteins, and/or lipids that are either enclosed or on their surfaces [4]. The International Society for Extracellular Vesicles has classified EVs into two subtypes: small (sEVs) and large extracellular vesicles (lEVs) [14]. Although recent studies have parsed EVs into categories including exosomes, ectosomes, microvesicles, apoptotic bodies, oncosomes, etc., we will concentrate on the sEV and lEV subgroups [2], both of which are further identified by their size, origin, contents, and functions [11].

Small EVs range between 30 and 150 nm in diameter, originate from the endosomal system, and involve late endosomal trafficking mechanisms [11]. sEVs are formed as intraluminal vesicles (ILVs) in intracellular endosomal multivesicular bodies (MVBs) and are released once MVBs fuse with the plasma membrane [4,15]. Actin-binding protein Cofilin1, enzymes such as Enolase1, Aldolase A, PGK1, and LDHA involved in cell metabolism, and heat shock proteins including Hsp90 and Hsp70 have all been identified as protein contained in sEVs from bladder tumors [16,17]. Certain tetraspanins, such as CD37, CD81, and CD82, are also highly enriched in sEV membranes [18]; CD63 seems to be also largely expressed in sEVs [19].

Large EVs are generally larger than sEVs with a diameter of 100 nm^−1^ µm and originate from the outward budding of the plasma membrane [11]. The lEVs are potentially involved in cell-cell communication and can participate in altering recipient cells [11]. Tetraspanin CD9 is mainly observed in large vesicles [15]. Studies of lEVs reveal that changes in phospholipid distribution in the plasma membrane lead to phosphatidylserine and phosphatidylethanolamine in the extracellular layer, which may induce shedding of lEVs [17]. Studies have found that cancer-derived lEVs are enriched in ADP-ribosylation factor 6 (ARF6) which was shown to trigger the shedding of EVs from breast cancer and prostate cancer cell lines [17,20].

Despite the lack of full understanding of EV formation, release, and uptake molecular mechanisms, studies have shown that various signaling molecules tightly regulate these processes [2].

### 1.4. EV Isolation

While there is not one standardized technique for EV isolation, a variety of techniques have been established and utilized to explore EV features [21]. There are many important factors to consider when isolating EVs depending on the starting material, whether media from cultivated cells, biological fluids, or tissue.

sEVs are generally isolated by ultracentrifugation-based methods as the gold standard, however, alternative methods have been developed based on factors such as EV precipitation and size [11]. Differential ultracentrifugation involves centrifugation steps to remove large vesicles, debris, and cells while precipitating sEVs [22]. While differential ultracentrifugation is a fairly standard procedure, the process requires large volumes of samples and is time-consuming [11]. Another method is density gradient centrifugation, in which the separation of EVs based on size and density is combined with a sucrose or iodixanol density gradient [22]. Density gradient centrifugation is effective in separating low-density sEVs from other particles, especially from bodily fluids. However, it is highly sensitive to centrifugation time and has low EV recovery [11,22].

Size exclusion chromatography (SEC) separates sEVs from other EVs by eluting substances out based on particle size [21]. This method results in high purity, quick preparation, good reproducibility, and low cost [2,21]. Still, size exclusion chromatography has low yield and low sample throughput, and filters used may cause deformation of EVs [2]. Immunoaffinity capture-based techniques use an antibody for a specific antigen to seize EVs based on the antigen expression on the EV surface [2]. Such techniques are efficient and lead to high purity with minimal processing time but result in low yield [2].

In the ELISA-based separation method, the antibody is bound to the microplate surface to isolate all sEVs and can be applied for quantification and characterization [11,22]. However, ELISA is not applicable for large sample volumes [22].

Microfluidics-based techniques are based on various principles such as size, density, and immunoaffinity [21]. While cost-effective, portable, and highly efficient, microfluidics-based techniques have low sample capacity [21]. Only one study has applied the use of a multilayer micro-nanofluidic device to isolate liposarcoma-derived EVs. This device integrated both size-based isolation and CD63 antibody immunoaffinity-based EV capture from conditioned media and patient serum [23]. This device allowed for around 32% recovery of EVs in DDLPS patient blood serum and around 76% of EVs in liposarcoma-derived Lipo246 cell line conditioned media. Using this device, processing time dropped nearly 85% compared to ultracentrifugation and EV cargo was preserved [23].

In addition to microfluidic devices, other methods to isolate sarcoma-derived EVs use microchips. Zhang et al. developed a microwell-patterned microfluidic platform with a dual-probe hybridization assay to detect specific mRNAs in EVs [24]. This platform was the first to quantitatively measure EWS-FLI1 mRNA copy numbers in EVs derived from Ewing’s Sarcoma [24]. However, this platform is not intended for the specific enrichment of EWS-EVs or retrieving EVs for downstream functional studies [25]. On the other hand, a recent study reports the development of a purification platform (ES-EV Click Chip) by coupling a covalent chemistry-mediated EV capture and release within a nanostructure-embedded microchip for EWS-derived EVs [25]. This device can be used for conducting downstream functional studies as it is capable of the precise and high purification of intact EWS-EVs [25].

Sequential use of multiple isolation techniques has been shown to greatly improve lipoprotein and protein contaminant depletion but may significantly reduce EV yield [26]. Appropriate isolation methods are chosen based on the initial quantity of material and the targeted amount of EVs [26]. The type of methodology used to isolate EVs can influence the resulting EV population, and thus impact the results. Thus, this review specifies the isolation methodology used for each study mentioned.

### 1.5. Contents of EVs

EVs contain cargos such as lipids, nucleic acids, proteins, and entities involved in lipid metabolism [4]. Packaged nucleic acids include small interfering RNA (siRNA), microRNA (miRNA), messenger RNA (mRNA), long non-coding RNA (lncRNA), DNA, mtDNA (mitochondria DNA) [4].

EV cargo also involves function-changing molecules such as adhesion molecules (e.g., integrins), chaperones, membrane trafficking molecules (e.g., Rab proteins), structural cytoskeleton molecules (e.g., actin and tubulin), and cytoplasmic enzymes (e.g., GAPDH) [27,28,29]. EVs also contain different types of proteins possibly involved in signal transduction pathways (e.g., kinases and phosphatases) and/or multivesicular body formation (e.g., clathrin) [5,30,31], molecules that are crucial for proper cell function and survival [4].

### 1.6. Function of EVs in Cancer

The functions for extracellular vesicles (EVs) include cell-to-cell communication, maintaining homeostasis between cells and tissues, and transfer of their cargos to the environment outside of the cell [32]. EV roles communicating their cargos to other cells can impact functions such as proliferation, apoptosis, [33] and other components of tumorigenesis, progression, and metastasis of various cancer types [2].

#### 1.6.1. Tumorigenesis

Tumor-derived EVs can influence normal stromal fibroblasts to become activated cancer-associated fibroblasts (CAFs) [34]. Prostate cancer-derived EVs can promote the conversion of normal fibroblast to CAF-like phenotype [35]. After CAFs are activated, they release EVs and cause an increase in cell proliferation and metabolic changes leading to tumorigenesis [36]. In another study, EVs released from ovarian cancer cells were able to activate the fibroblasts and transform them into CAFs [36]; EVs from a more aggressive ovarian cancer cell (SKOV3) were more successful in this activation process and promoted tumorigenesis than EVs derived from less aggressive cell type (SKOV3) [36].

Another study investigated the role of breast cancer derived-EVs in promoting tumorigenesis [37]. The study also demonstrated that cancer derived EVs contain proteins necessary for miRNA synthesis, including Dicer [37]. This study explored how miRNA biogenesis contributes to the progression of cancer by comparing the miRNA profile of cancer cell lines after they were treated with EVs containing Dicer antibodies [37]. Inhibiting the function of Dicer in cancer derived-EVs, reduce the growth of tumors, suggesting that EV miRNA plays a role in tumorigenesis [37].

#### 1.6.2. Progression/Proliferation

EVs also play a key role also in regulating cancer progression through their cargos [38]. Several studies have shown that after the resection of tumorous tissues, many miRNA levels as EV biomarkers return to normal [39,40,41], suggesting that there may be a link between the sorting of miRNA into EVs and the progression of cancer.

To determine the effects of EV on gastric cancer cells, gastric cancer cells were treated with conditioned media from acute monocytic leukemia cell line, observing that the treated cells promoted the proliferation and invasion of gastric cancer cells. This effect was due to the activation of macrophages and other heterogeneous cells that subsequently promote tumor growth and metastasis in the tumor microenvironment, including tumor cell migration and proliferation. The EVs upregulated the phosphorylation of NF-kB pathway in macrophages, leading to increased expression of proinflammatory factors in THP-1 cells. Furthermore, it was found that inhibiting NF-kB reversed the role of cancer EVs in the progression of gastric cancer [42].

Corrado et al. hypothesized that EVs played a functional role in the bidirectional crosstalk between the bone marrow stromal microenvironment and cancer cells, leading the progression of chronic myelogenous leukemia (CML) [43,44]. After treating bone marrow stromal cells with EVs derived from CML, they found an increase in mRNA and interleukin-8 (IL8) protein expression. IL8 is a pro-inflammatory chemokine that activates many signaling pathways downstream of two domain receptors, CXCR1 and CXCR2 [45]. Taken together, this study shows that EVs secreted from CML cells are capable of stimulating bone marrow stromal cells to generate IL8, which in turn stimulates leukemia cell growth thereby contributing to its progression [43].

Another study provided evidence of the role of EVs from colon cancer sources leading to a tumor-like transformation of mesenchymal stem cells (MSCs). Colorectal cancer cell EVs prompt increased proliferation, migration, and invasion in cMSCs leading to cancer progression and a functional 50% increase in cMSC proliferation. This prevalent increase in cell proliferation resisted for seven days after the removal of the human primary colorectal carcinoma cell line (pCRC), demonstrating that tumor-derived EVs can induce persistent proliferation in cMSCs. In conditions stimulating the tumor microenvironment, such as low pH and low serum, an additional 50% increase was found in cMSC proliferation. This rate of proliferation for cMSCs treated with EVs is similar to the rate of proliferation in colorectal cancer cells, with up to a 6-fold increase in cell migration and a 2.4-fold increase in cell invasion. Such results clearly suggest the potent role of EVs in inducing cMSC malignant behavior [46].

#### 1.6.3. Metastasis

EVs also appear to play a central role in metastasis. Hood et al. uses a tumor-derived EV-dependent model of lymphatic metastatic progression to investigate the hypothesis that preconditioned regional or sentinel lymph nodes are involved in metastasis progression. Melanoma EVs are capable of recruiting melanoma cells to sentinel lymph nodes, but in the absence of melanoma cells, melanoma EVs induce sentinel nodes. These nodes then promote the expression of interconnected extracellular matrix factors, which may lead to melanoma cells confined in sentinel nodes. Melanoma EVs present in lymph nodes prompt the induction of angiogenic growth factors that are needed for melanoma growth. Previous studies have shown that melanoma EVs stimulate endothelial spheroid production in vitro; tumor EVs can induce paracrine endothelial signaling [47]. These results showed that melanoma use EVs as a means to induce site preparation for metastasis through the lEV premetastatic conditioning of lymph nodes [48].

EVs secreted by tumor stroma can also impact tumor progression and metastasis [49]. Luga et al. reports that fibroblasts associated with breast cancer secrete EVs that advance breast cancer cell motility, protrusive activity, and invasion through Wnt-planar cell polarity (PCPC) signaling. Mouse models of breast cancer were coinjected with breast cancer cells (BCCS) and fibroblasts, leading to increased metastasis dependent on EV cargo CD81 in fibroblasts and Wnt-planar cell polarity (PCP) signaling in BCCs [50].

EVs have the ability to increase the metastatic potential of malignant tumor cells. In a study that uses the Cre-LoxP system to recognize tumor cells that uptake EVs in vivo, it has been shown that highly metastatic tumor cells can transfer biomolecules such as Cre to less malignant cells. The less malignant cells that uptake the EVs demonstrated increased migration and metastatic capacity, as displayed in intravital imaging [51].

### 1.7. Therapeutic Applications of EVs

Extracellular vesicles have a myriad of potential therapeutic applications involving cancer treatments. Firstly, numerous EVs and their cargo are potential biomarkers of cancer development and tumor advancement, leading to an increase in personalizing cancer diagnoses [52]. Furthermore, EV miRNA expression is an indicator of metastasis, as studies have revealed many miRNAs (miR-9, miR-10b, and miR-182) involved in increased metastasis [53]. Therefore, miRNA testing in the EVs of patients can resemble a marker used to forecast and indicate the metastatic potential of a tumor [53]. Another example of the implications of EVs includes their role as a delivery tool. Because EVs are abundant in bodily fluids and can traverse biological barriers, such features have stimulated efforts to understand and advance the potential for EVs as a drug-delivery system to distribute chemotherapeutics, miRNAs, anti-miRNAs, and much more [54]. This therapeutic application has been studied in animal models and in vitro [55,56].

Other possible therapeutics are the EVs derived from mesenchymal stem cells (MSC) as in one study, MSC-EVs were injected weekly into rats immunocompromised rats resulting in increasing the formation of neocartilage, hyaline cartilage, and regeneration of subchondral bone after 12 weeks [57]. Modifying the contents of EVs such as filling them with therapeutic proteins or RNAs that could be transported to the recipient cells was also demonstrated in developing therapeutic EVs [58]. One study engineered the therapeutic EVs to express a specific protein, Lamp2b, that could enhance the biological function in the cells [59]. The discussion of vesicles as therapeutic methods is not the focus of this paper. Please refer to this review by Wiklander et al. for further information [58].

## 2. EVs in Sarcoma

### 2.1. EVs in Ewing Sarcoma

Ewing Sarcoma (EWS) is a highly aggressive bone sarcoma; it is the second most common malignant bone tumor in children and adolescents after osteosarcoma [60]. The main molecular derangement driving EWS is a translocation between the EWSR1 gene in chromosome 22 and chromosome 11 [61]. This results in the EWS/FLI-1fusion gene which is a product of the translocation t(11;22) (q24; 12) and is detected in 95% of EWS patients [62].

EVs were first identified in 2013 in Ewing sarcoma cell lines [63]. A gene-set enrichment analysis (GSEA) was conducted, showing that EWS EVs contained cargo that involves neurotransmitter signaling and G-protein-coupled signaling [63]. Also, mRNAs that are generally associated with EWS tumorigenesis were found as EV cargo, including EWS/FLI-1 [63]. Further research on EWS/FLI-1as EV cargo demonstrated that EWS-derived EVs could transfer EWS-FLI1 mRNA to other EWS cells but not to osteosarcoma cells, implying that EVs may be involved in EWS cell cross-talk resulting in more tumorigenic states [62,64]. A separate study investigated the role of CD99 in EWS oncogenesis, revealing that EWS cells where CD99 is silenced, release EVs having high level of miR-34a; the delivery of this EVs to EWS recipient cells induced miR-34 inhibition of Notch-NFkB signaling and drove neural differentiation [65].

Further research showed that since CD99 expression was equivalent in EWS cells with or without CD99neg EVs, regulation of the cell behavior was likely independent of CD99 presence and dependent on EV cargo [66]. miR-199a-3p was the most enriched miRNA cargo found in CD99neg EVs and acts through the activator protein-1 signaling pathway. Because miR-199a had higher levels of expression in localized tumors compared to metastasis, this cargo may contribute more to the aggressiveness and resistance of EWS. Taken together, both miR-199a-3p and miR-34a are found in CD99neg EV cargo, functioning to reduce cell growth and migration and stimulate neural differentiation [66].

### 2.2. EVs in Rhabdomyosarcoma

Rhabdomyosarcoma (RMS) is the most common soft tissue sarcoma in children. There are two major biologically distinct types of RMS identified: embryonal and alveolar. Patients with embryonal RMS (ERMS) are different from those with alveolar RMS (ARMS) regarding age of onset, tumor site, and long-term outcome [67]. Furthermore, a worse prognosis for this disease is associated with the presence of PAX3-FOXO1 fusion oncoprotein. Most patients with localized rhabdomyosarcoma can be treated using combinations of radiation, chemotherapy, and surgical resection; however, patients with metastatic rhabdomyosarcoma still respond poorly to the treatments [68].

Rammal et al. isolated EVs from five RMS cell lines and performed a proteomic characterization. They found 80 common proteins as cargo [69]. Of those proteins, 28% were involved in cellular processes (such as cell communication, cell cycle, cellular movement), 16% were involved in metabolic processes, and the remainder were components of cellular organization, localization, and immune system processes [69]. Among those proteins, they also identified molecules not been previously found in other EVs. These molecules are specific to RMS-EVs, such as BMP1, CDKN2A, or ITGA7, that are involved in cell proliferation, migration, and invasion. Another study showed that EVs isolated from both RMS subtypes (embryonal and alveolar) contained miR-1246 and miR-1268 as cargo in all RMS EVs evaluated [70]. EV miR-1246 and miR-1268 increased the migration and invasion of normal fibroblasts and endothelial cells [70]. These miRNAs play a role in Integrin and p53/Ras pathways, suggesting their involvement in proliferation, angiogenesis, and metastasis. What the EVs derived from the fusion-negative RMS cell lines had in common was the enrichment of miRNA related to cancer progression, including Cyclin proteins (Cyclin D1, IGF, AKT, SP1, and CDKN2A), and proteins involved in cell proliferation (NFYB), invasion (YBX1), and cell transformation (HMGA1) [70]. As for fusion-positive EVs, miRNA implicated in cancer, inflammation, connective tissue diseases, and angiogenesis have found to be increased (MDM2, CDKN1A, CDKN2A, IGF1R, SOX2, YBX1, BRINP3).

An additional study demonstrated an important role for PAX3-FOXO1 in modulating the EV content of myoblasts through its impact on miR-486-5p [71]. Data from this study showed that PAX3-FOXO1 influenced the miRNA EV content [71]. While PAX3-FOXO1 protein is not included in EVs, it seems that PAX3-FOXO1 transduced cells, activated transcription of miR-486-5p through its binding to the upstream sANK1 promoter [72]. Enhance of miR-486-5p resulted in pro-tumorigenic recipient cells (fibroblast, C2C12) which exhibited increased proliferation, migration, invasion, and colony formation [71]. The putative downstream candidate targets of miR-486-5p responsible for such behavior have been identified as Trp53inp1, Smad2, Cdkn2b, Pdgfrβ, and Pim1. Although previously identified as a tumor suppressor, miR-486-5p acts as an oncogenic simulant in RMS and has been shown to be at higher levels in the tumor-derived EVs of RMS patients.

### 2.3. EVs in Kaposi’s Sarcoma

Kaposi’s sarcoma (KS) is the most common AIDS-defining cancer in the world [73], and is caused by Kaposi’s sarcoma-associated herpesvirus [74]. Due to the recent focus on EVs as a potential contributor to oncogenesis, many studies have sought to determine the role of EVs in KS and their cargos. KS EV cargo derived from serum of KSHV-positive mice contained high levels of KSHV-encoded miRNAs that are members of the miR-17-92 cluster [73].

One study collected EVs from cell culture conditioned media to reveal that KSHV infection modified the proteins and microRNAs in the EVs released from KSHV-infected B-cells [75]. KSHV codes for a complement inhibitor that can suppress the complement system. However, EVs activate the complement system during KSHV infection through the activation of endogenous C3 and properdin [75]. KSHV activation leads to activation of the complement system in both the infected cells and the neighboring endothelial cells. This EV-mediated complement activation during KSHV may be considered as an immune response and may explain why KS is an inflammatory tumor.

Another study that examined EV-mediated pathways in KS determined that although EVs released from infected cells do not lead to infection or replication, but they detrimentally influence the immune response of the host. The quantity of EVs released form KSHV-infected cells has found to be increased. This has been proposed to lead to the increase of released mitochondrial DNA, and so affect antiviral defenses [75]. Singh et al. detected IFI16 and cleaved IL-1β in the EVs released from BCBL-1 cells. They proposed that this release could be a strategy to impact IL-1β functions and induce immune evasion [76]. It is also known that KSHV produce viral miRNAs that can induce changes in gene expression of the host and promote the virus to remain in the individual. B cells EVs have been influenced in their behavior from EVs released from infected cells. For example, a cargo of KSHV-infected cells are proteins involved in glycolytic metabolisms [77]. Other EV protein cargos, such as viral lytic proteins, also influence host endothelial cell movement and anchorage [74], implying that EVs from KSHV-infected cells can influence surrounding normal endothelial cells, perhaps via cell cycle and immune system pathways [77].

### 2.4. EVs in Osteosarcoma

Osteosarcoma (OS) is a bone cancer rare in adults but common in children. The leading cause of this disease is a mutation in bone cell DNA sequences; long-term survival rates depend on the disease stage and vary between 30 and 70% [32]. As with other cancer cells, OS cells communicate with their microenvironment through extracellular vesicles. EVs mediate crosstalk by transferring their cargo components osteoclasts, OS cells, fibroblasts, and endothelial cells [32]. The OS-derived EVs can trigger angiogenesis, osteoclastogenesis, and OS cell growth [32]. Major cargos in OS-derived EVs include growth factors such as transforming growth factor-beta (TGFβ), urokinase plasminogen activator (uPA), matrix metalloproteinases (MMP1 and MMP3), proteins such as RANKL, and miRNAs. Each of these cargos can contribute to the oncogenicity of osteosarcoma through different mechanisms.

Several studies have explored the role of EVs in primary bone cancer progression [78,79,80]. One study demonstrated that OS EVs were involved with bone remodeling. OS-derived EVs contribute a membranous form of transforming growth factor-beta (TGFβ) to mesenchymal stem cells (MSCs), which induces an increase in their releases of interleukin 6 (IL6) [78]. IL6 has a significant role as proinflammatory cytokine and is implicated in proliferation and cell cycle progression. Another study reported high expression of urokinase Plasminogen Activator (uPA) and its plasma membrane-associated receptor (uPAR) in OS cell lines and their EVs which correlated with lung metastatic behavior [79]. OS EVs shuttle uPA and uPAR to osteosarcoma target cells and help in the preparation of the metastatic niche. This was consistent with the reduction of metastatic activity by pharmacological or genetic inhibition of the uPA/uPAR axis in vivo [79]. Osteoclastic EV cargos such as MMP1 and MMP13, RANKl, TGFβ were detected on EVs isolated from conditioned media of human osteosarcoma cell cultures. These cargos have been hypothesized to dysregulate bone remodeling and increase osteoclastic activity in osteosarcoma [81].

Moreover, analysis of EV OS cell line revealed specific miRNA cargos that are associated with OS progression [82,83,84]. miR-195 was much lower in OS patient sera than healthy individuals [82]; miR-148a and miR-25-3p in EVs are higher respectively in peripheral blood and sera of OS patients, and their increase as EV cargos was associated with enhanced tumor growth [83,84].

The role of miRNA cargos in facilitating oncogenicity was explored in other studies in which a difference in miRNA content between metastasis and non-metastatic OS-derived EVs was observed [64,85,86,87].

One study identified mainly four miRNAs present in OS EVs (miR-143-3p, miR-21-5p, miR-181a-5p, and miR-148-5p) that potentially could target genes related to apoptosis [85]. miR-675 has been found also as a cargo of OS-derived EVs, and its uptake led to the downregulation of CALN1 expression in non-malignant fibroblast recipient cells influencing invasion and migration [86]. miR-21 is another OS-derived EV cargo that has a role in tumor progression. Patient with OS have been described to have higher levels of circulating miR-21. Studies have demonstrated that the presence of OS could be determined by the detection of plasma miR-21 together with miR-143 and miR-199a-3p. Additionally, miR-21 predicts poor prognosis in patients with osteosarcoma [88]. The mechanism by which miR-21 has such a role in OS, seems to be the inhibition of Apoptosis of Osteosarcoma via miR21-Targeting of Caspase 8 [89]. Lastly, another study demonstrated that OS-derived EVs such as miR-148a and miR-21-5p induce angiogenesis in endothelial cells and bone remodeling [87].

### 2.5. EVs in Liposarcoma

Liposarcoma (LPS) is the most prevalent soft tissue sarcoma histological subtype. It is classified into four subtypes: (i) well-differentiated LPS (WDLPS), (ii) de-differentiated LPS (DDLPS), (iii) myxoid/round cell LPS (MRC), and (iv) pleomorphic LPS. Among the four LPS subtypes, dedifferentiated liposarcoma (DDLPS) has propensity for local and distant recurrence and often recur as synchronous multifocal tumors.

The role of the EVs in liposarcoma has been studied in the two most common subtypes of liposarcoma: well-differentiated and dedifferentiated varieties. In these tumors, EV cargos may play an important role inducing tumor progression and metastasis. Major cargos in liposarcoma EVs include MDM2 DNA and specific miRNAs that may promote oncogenicity [90,91].

MDM2 DNA is located on chromosome 12. The region of the chromosome 12q13-15 is amplified in WD/DDLPS, and MDM2 amplification is recognized as the main driver of the disease. MDM2 amplification leads to MDM2 protein overproduction. MDM2 protein functions as a negative regulator of p53 [92,93]; after binding to p53, MDM2 inhibits its transcriptional activity, induces its nuclear export and its degradation [93]. Casadei et al.’s paper examined whether EVs derived from DDLPS patient blood or DDLPS cell lines could be potential carriers of MDM2 DNA. In this study, they first assessed EVs isolated from DDLPS patient serum for the level of MDM2 DNA compared to healthy controls. The results showed increased MDM2 DNA levels in DDLPS patient EVs when compared to normal ones. Second, they found that DDLPS cell-line-derived EVs had higher levels of MDM2 DNA compared to preadipocyte-derived EVs. Next, they incubated the preadipocytes (prevalent in the fat-bearing retroperitoneal compartment) with DDLPS-derived EVs and found an increased level of MDM2 at mRNA and protein level, which showed that MDM2 DNA was transferred from DDLPS-derived EVs to preadipocytes and translated into protein. Furthermore, when P-a that were exposed to DDLPS EVs, they undergo a reduction of p53 and p21 levels and exhibit enhanced proliferation and migration. The study further demonstrated that the transfer of EV-derived MDM2 DNA promotes the production of a metalloproteinase, MMP2, by preadipocytes, which favors pre-metastatic niches in DDLPS [94]. Hence, MDM2 as a cargo of DDLPS EVs could potentially lead to the recurrence phenomena in WD/DDLPS.

The other two cargos, miR-25–3p and miR-92a-3p, have been recognized to have a role in liposarcoma progression [95]. In one study they explored their mechanism in liposarcoma oncogenicity: they first isolated EVs from both liposarcoma cells and preadipocytes through ultracentrifugation and assessed the miRNA expression levels. Their results showed that the expression levels of two specific miRNAs, miR-25–3p and miR-92a-3, were higher in liposarcoma-derived EVs than preadipocytes. They then wanted to see whether these miRNAs were involved in the communication between the WD/DDLPS and the surrounding microenvironment. Through the ELISA assay, they found that miRNAs promoted the secretion of Interleukin 6 (IL-6), a pro-inflammatory cytokine, from macrophages. They also found that IL6 secretion induced by liposarcoma-derived EVs occurred in a TLR7/8-dependent manner, and they finally established that EV- stimulated secretion of IL6 promotes in turn liposarcoma proliferation, migration and invasion [95]. Hence, macrophage-secreted IL-6 stimulates the growth of tumors in TIME and promotes cell proliferation, migration, and invasion.

## 3. Conclusions

**Figure 1:**
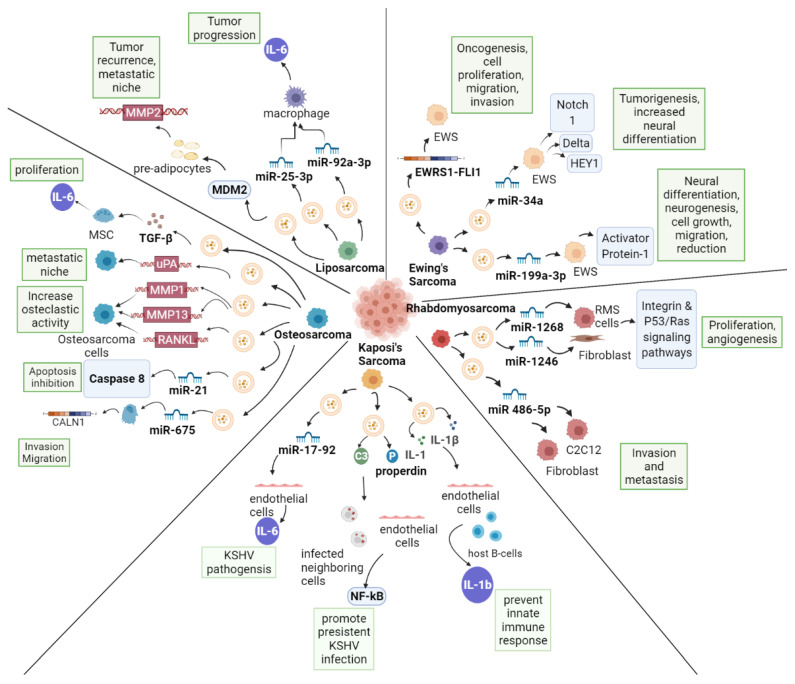
EVs in Sarcoma.

EVs release various cargos in different sarcomas (i.e., miRNA, proteins or DNAs, Table 1) and can affect the recipient cell phenotype and the aggressivity of the tumor itself, via multiple mechanisms (Figure 1). The study of EV in sarcoma, is still limited and further research may help establish novel therapeutic approaches that target specific sarcoma subtypes or biologies, thereby improving sarcoma therapeutics in the future.

## Figures and Tables

**Table 1:** EVs in Sarcoma. The table details each type of sarcoma, their EV cargos, the targets of specific cargos, and their function.

Sarcoma Type	EV Cargo	Function	Targets	References
Ewing’s Sarcoma	EWS/FLI-1	Oncogenesis	NA	[62,63]
	miR-34a	Promotion of neural differentiation	Notch/NF-kβ pathway	[65,66]
	miR-199a-3p	Promotion of neural differentiation	Activator protein-1 signaling pathway	[66]
Rhabdomyosarcoma	BMP1, CDKN2A, ITGA7	Proliferation, invasion, migration	NA	[69]
	miR-1246, miR-1268	Proliferation, angiogenesis, metastasis	Integrin and p53/Ras	[70]
	miR-486-5p	Proliferation, invasion, metastasis	Putatively Trp53inp1, Smad2, Cdkn2b, Pdgfrβ, Pim1	[71]
Kaposi’s Sarcoma	miR-17-92	Cell migration, IL6 secretion	NA	[73]
	C3 and properdin	Promotes persistent KSHV infection	NF-kβ pathway	[75]
	IL-1, IFI16	Prevent innate immune responses	Remove host factors (i.e., IL-1b)	[76]
Osteosarcoma	TGFβ	Proliferation	IL-6 production-MSCs	[78]
	uPA, uPAR	Metastatic niche	NA	[79]
	MMP1 MMP13 RANKL	Increase osteoclastic activity	NA	[81]
	miR-21	Inhibition apoptosis	Caspase 8	[88,89]
	miR-148a miR-21-5p	Angiogenesis Bone remodeling	NA NA	[87]
	miR-675	Invasion, migration	CALN1	[86]
Liposarcoma	MDM2	Proliferation, migration, tumor recurrences	MMP2	[95]
	miR-25-3p miR-92a-3p	Stimulate tumor progression (enhanced proliferation, migration, invasion)	TLR7/8, IL6	[94]

## Data Availability

Not applicable.
